# Microsurgical Resection of Meningiomas Using a 4K Three-Dimensional Exoscope: A Descriptive Observational Study

**DOI:** 10.7759/cureus.74950

**Published:** 2024-12-02

**Authors:** Toshiaki Kodera, Makoto Isozaki, Satoshi Kawajiri, Shinsuke Yamada, Takahiro Yamauchi, Hidetaka Arishima, Kenichiro Kikuta

**Affiliations:** 1 Department of Neurosurgery, University of Fukui, Fukui, JPN

**Keywords:** case series, exoscope, meningioma, microscope, neurosurgery

## Abstract

Background

Prior to using the exoscope, we speculated that it represented an intermediate tool between a loupe and a microscope and had concerns about its visibility of deep, fine structures.

Objective

To evaluate the depths of meningioma for which the exoscope was suitable, and to clarify its disadvantages in meningioma resection.

Methods

Findings of consecutive meningioma surgeries using a 4K three-dimensional (3D) exoscope over a one-year period were evaluated for visibility of the surgical field, comfort of the surgeon’s arm posture, the surgeon’s head orientation, and perception of the image delay, accounting for the depth of the tumor.

Results

Seven meningiomas were resected using a 4K 3D exoscope (three superficial, three intermediate, and one deep). The exoscope allowed the surgeon to observe deeply located fine structures as clearly as with a conventional microscope and to operate more comfortably on meningiomas of all depths with arms flexed. On the contrary, the exoscope occasionally required the surgeon to operate with his head unnaturally turned to one side because of the immobility of its large monitor, despite the wide insertion availability of its camera from various directions to meningiomas located superficially or within the middle cranial fossa. No time delays between the surgeon’s manipulations and the 3D images were perceived in all meningioma surgeries.

Conclusions

The 4K 3D exoscope was suitable for operations on all depths of meningiomas. The discrepancy between the surgeon’s manipulation and gaze directions was its disadvantage. It is anticipated that further development of the 3D monitor will address this issue.

## Introduction

Since the 1960s, the surgical microscope has been an essential tool for performing a range of neurosurgical operations, including the resection of meningiomas. More recently, an exoscope has been developed and is being increasingly used to perform microneurosurgical operations as an alternative to the traditional microscope. The exoscope can be considered a “halfway instrument” between a microscope and an endoscope. When using an exoscope, surgeons stand facing the surgical field with a three-dimensional (3D) camera and perform microsurgery while watching a front-facing monitor with the assistance of 3D glasses [1-4]. A novel 4K 3D exoscope is compact and can clearly display surgical fields on one or two monitors with 4K resolution imaging quality [5-10].

The University of Fukui Hospital (UFH) began using the 4K 3D exoscope for various neurosurgical procedures in 2021. We anticipated that the exoscope would function as both a loupe and a conventional microscope, despite concerns raised by several neurosurgeons about the visibility of deep structures [11,12]. Based on our experiences with several cases of meningioma resection using the 4K 3D exoscope, we aimed to evaluate its advantages and disadvantages compared with those of a conventional microscope, as well as the depths of the lesion for which exoscopic surgery was suitable.

## Materials and methods

Meningioma surgery using a 4K 3D exoscope

**Figure 1:**
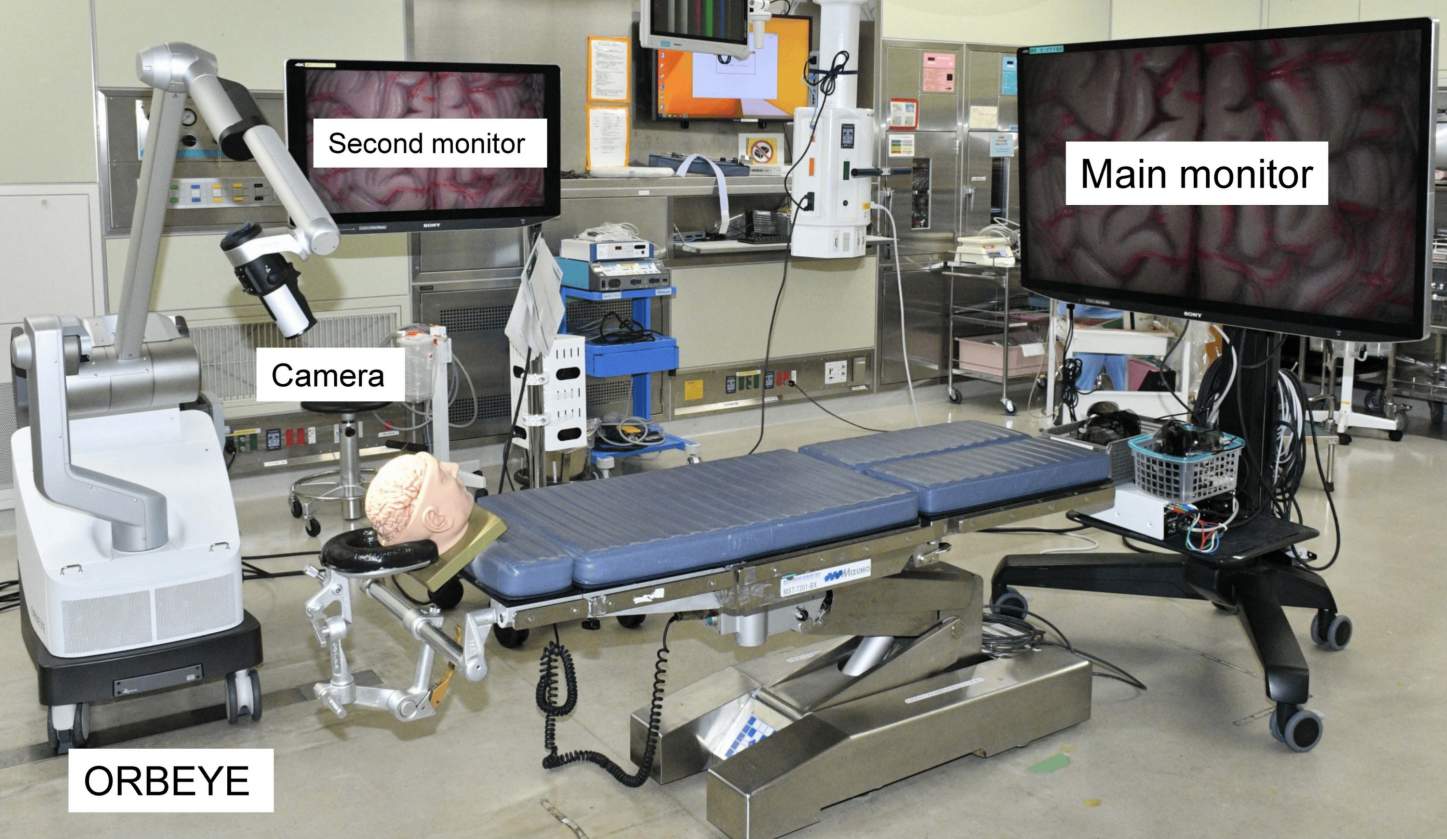
Operating room setup for the 4K three-dimensional exoscope system. A main large monitor for the primary surgeon is placed at the foot of the operating table.

Consecutive meningioma surgeries, which were performed by experienced neurosurgeons at UFH from March 2021 to February 2022, were evaluated. In all surgeries, a novel 4K 3D exoscope (ORBEYE; Sony Olympus Medical Solutions, Tokyo, Japan) was used instead of a conventional microscope; patients were placed in the supine position; a main large 4K 3D monitor was placed at the foot of the operating table (Figure 1); and the primary surgeon performed the meningioma resection while watching the main monitor through 3D glasses. An assistant surgeon supported the surgery by observing the main monitor or a second small 4K 3D monitor placed beside the patient using 3D glasses. The exoscope was used from the time of the dural incision to dural closure during surgery. The patients consented to the procedure, and the participants and any identifiable individuals consented to the publication of their images.

Meningioma classification according to depth

According to the depth of the tumor, which was defined as the distance between the brain surface and the dural attachment of the tumor, meningiomas were classified into three groups: meningiomas attached to the superficial dura were classified as “superficial”; meningiomas attached to the falx, tentorium, or skull base dura, without attaching to the superficial dura and without involving the cranial nerves or major arteries, were classified as “intermediate”; and meningiomas attached to the deep part of the falx, tentorium, or skull base dura, with involvement of the cranial nerves or major arteries, were classified as “deep”.

Evaluation of the exoscopic surgery

For each surgery, the following four concerns were evaluated: (1) visibility of the tumor and surrounding structures; (2) comfort of the surgeon’s arm posture; (3) surgeon’s head orientation; (4) perception of the time delay between the surgeon’s manipulations and 3D images on the monitor. Scores of +1, 0, or -1 were given based on whether the surgeons considered the exoscope superior, equivalent, or inferior to the conventional microscope just after each surgery, respectively. The mean scores for each tumor depth were summed for each depth or concern; the exoscope was considered superior to the conventional microscope when the total score was +0.5 or higher, equivalent when it was higher than -0.5 and lower than +0.5, and inferior when it was -0.5 or lower. These total scores were then used to determine the depths of meningioma for which the exoscope was suitable for resection, as well as the advantages and disadvantages of the exoscope in meningioma surgery.

## Results

**Figure 2:**
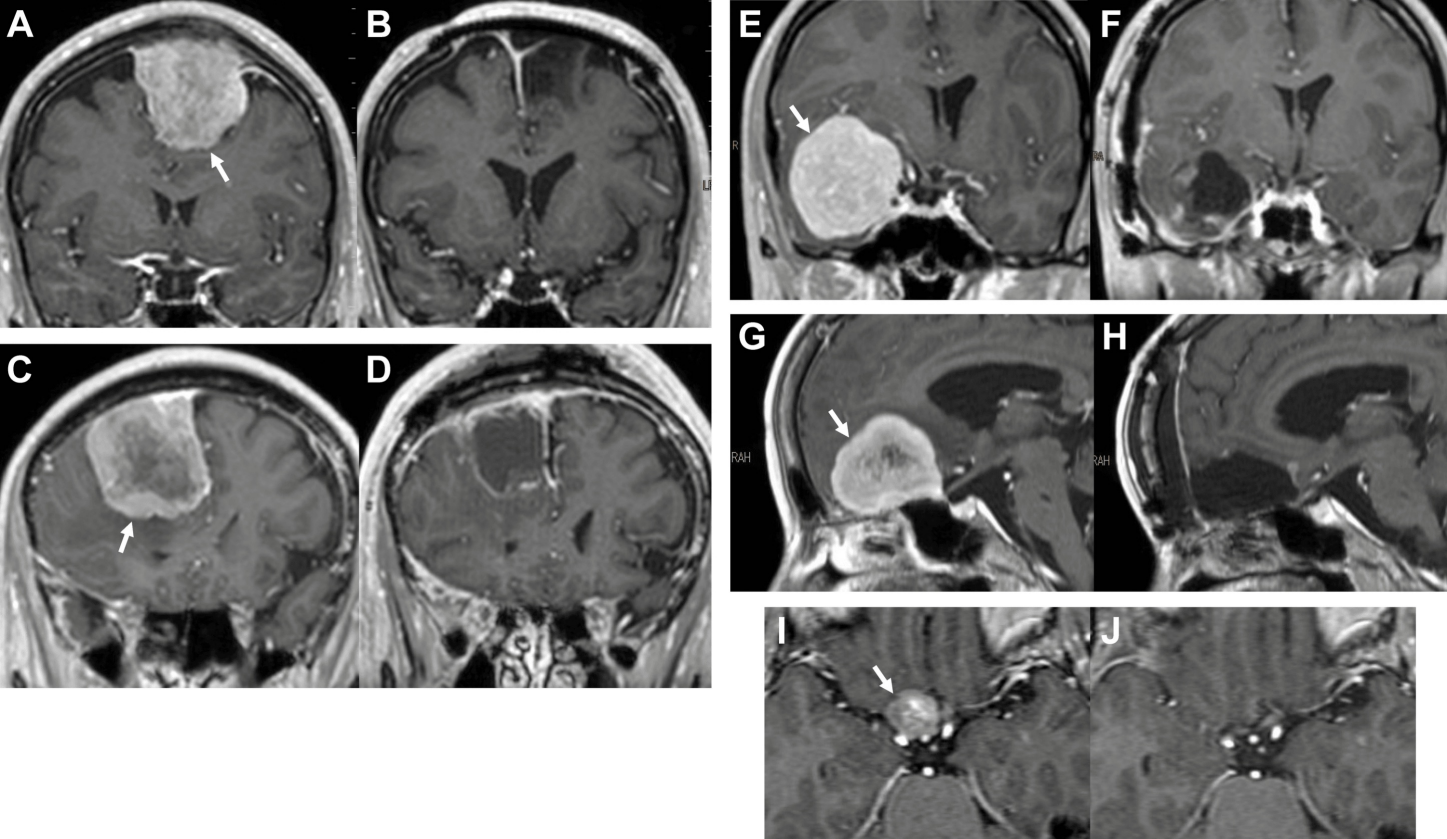
Representative magnetic resonance images (gadolinium-enhanced T1-weighted images). (A)-(B) Before and after surgery of case 2 in the presence of a left convexity-parasagittal meningioma (arrow). (C)-(D) Before and after surgery of case 3 in the presence of a right parasagittal-falx meningioma (arrow). (E)-(F) Before and after surgery of case 5 in the presence of a right middle fossa meningioma (arrow). (G)-(H) Before and after surgery of case 6 in the presence of an anterior fossa meningioma (arrow). (I)-(J) Before and after surgery of case 7 in the presence of a right tuberculum sellae meningioma (arrow).

Seven meningiomas were resected using the 4K 3D exoscope during the study period. Three were superficial (one convexity, one convexity-parasagittal, one parasagittal-falx); three were intermediate (two middle fossa, one anterior fossa); and one was deep (one tuberculum sellae). Characteristics of the seven patients are presented in Table 1, and representative magnetic resonance images of the meningiomas are shown in Figure 2. Five patients had presurgical subjective symptoms, one had papilledema found on medical checkup, and one had no symptoms other than paranasal sinusitis. Presurgical Karnofsky Performance Status (KPS) was 60% in one patient, 70% in one patient, 80% in three patients, and 100% in two patients. Gross total resection was achieved in all patients (Simpson grade 1 in two, grade 2 in five). The mean follow-up period after surgery was 28.4 months. All symptoms, except anosmia caused by an anterior fossa meningioma, were improved after surgery. Postoperatively, the KPS became higher in five patients and remained unchanged (at 100%) in two patients. One patient with a tuberculum sellae meningioma developed a surgical complication in the form of a right olfactory nerve injury; however, the presurgical symptom of right visual impairment was improved postoperatively.

**Table 1:** Characteristics of patients with a meningioma resected using a 4K 3D exoscope. 3D, three-dimensional; F, female; KPS, Karnofsky performance status; Lt, left; M, male; mo, months; Rt, right

Case	Age in years /sex	Before surgery				Surgery		After surgery			
		Symptoms	KPS	Tumor site	Tumor size (mm)	Approach	Simpson grade	Complications	Presurgical symptoms	KPS	Follow up (mo)
1	80/M	Aphasia, Rt hemiparesis	60%	Lt convexity	58 x 46 x 50	Fronto-temporal craniotomy	Grade 1	None	Improved	100%	29
2	46/F	Papilledema on medical checkup	100%	Lt convexity- parasagittal	48 x 42 x 44	Frontal craniotomy	Grade 2	None	Unchanged	100%	25
3	62/M	Lt hemiparesis	80%	Rt parasagittal- falx	59 x 42 x 50	Frontal craniotomy	Grade 2	None	Improved	100%	35
4	82/F	Dementia	70%	Lt middle fossa	50 x 52 x 55	Zygomatic approach	Grade 2	None	Improved	100%	25
5	39/F	No symptom (paranasal sinusitis)	100%	Rt middle fossa	47 x 46 x 47	Orbitozygomatic approach	Grade 2	None	Unchanged	100%	24
6	65/F	Anosmia, memory/emotion disturbance	80%	Anterior fossa	40 x 40 x 33	Fronto-basal approach	Grade 1	None	Improved except anosmia	90%	28
7	51/F	Rt visual impairment	80%	Rt tuberculum sellae	16 x 17 x 10	Transsylvian approach	Grade 2	Rt olfactory nerve injury	Improved	90%	33

“Head-up position” surgery was easily accepted by the surgeons, who were familiar with the conventional microscope. Table 2 shows the results (scores) of the visibility of the surgical field, comfort of the surgeon’s arm posture, surgeon’s head orientation, and perception of the image delay during meningioma resections using the exoscope, compared with those using the conventional microscope for each tumor depth.

**Table 2:** Evaluation of the 4K 3D exoscope compared to the conventional microscope depending on the depth of tumor. For each meningioma surgery, each concern (visibility of the surgical field, comfort of the surgeon’s arm posture, surgeon’s head orientation, and perception of the image delay) was evaluated and scored according to whether the primary surgeon considered the 4K 3D exoscope superior (+1), equivalent (0), or inferior (-1) to the conventional microscope. The mean scores of each tumor depth were summed for each depth or concern. Based on the results, the exoscope was considered superior to the conventional microscope when the total score was +0.5 or higher, equivalent when it was higher than -0.5 and lower than +0.5, and inferior when it was -0.5 or lower. The results tabulated in this table indicated that the 4K 3D exoscope was suitable for all depths of meningiomas, and its sole disadvantage was the surgeon’s unnatural head orientation. *, During superficial meningioma resection, wider surgical field could be observed than that when using the conventional microscope; †, Deeply located fine structures could be observed as clearly as that when using the conventional microscope; ‡, A convexity meningioma could be resected without extension of the surgeon’s arms even when using the conventional microscope. 3D, three-dimensional; Lt, left; Rt, right

Tumor depth	Tumor site	Visibility of surgical field	Surgeon’s arm posture	Surgeon’s head orientation	Perception of image delay	Total score
Superficial	1) Lt convexity	+1*	0‡	-1	0	+0.67 (superior)
	2) Lt convexity-parasagittal	+1*	+1	-1	0	
	3) Rt parasagittal-falx	+1*	+1	-1	0	
		Mean, +1	Mean, +0.67	Mean, -1	Mean, 0	
Intermediate	4) Lt middle fossa	0	+1	-1	0	+0.33 (equivalent)
	5) Rt middle fossa	0	+1	-1	0	
	6) Anterior fossa	0	+1	0	0	
		Mean, 0	Mean, +1	Mean, -0.67	Mean, 0	
Deep	7) Rt tuberculum sellae	0†	+1	0	0	+1 (superior)
Total score		+1 (superior)	+2.67 (superior)	-1.67 (inferior)	0 (equivalent)	+2 (superior)

Visibility of the surgical field

**Figure 3:**
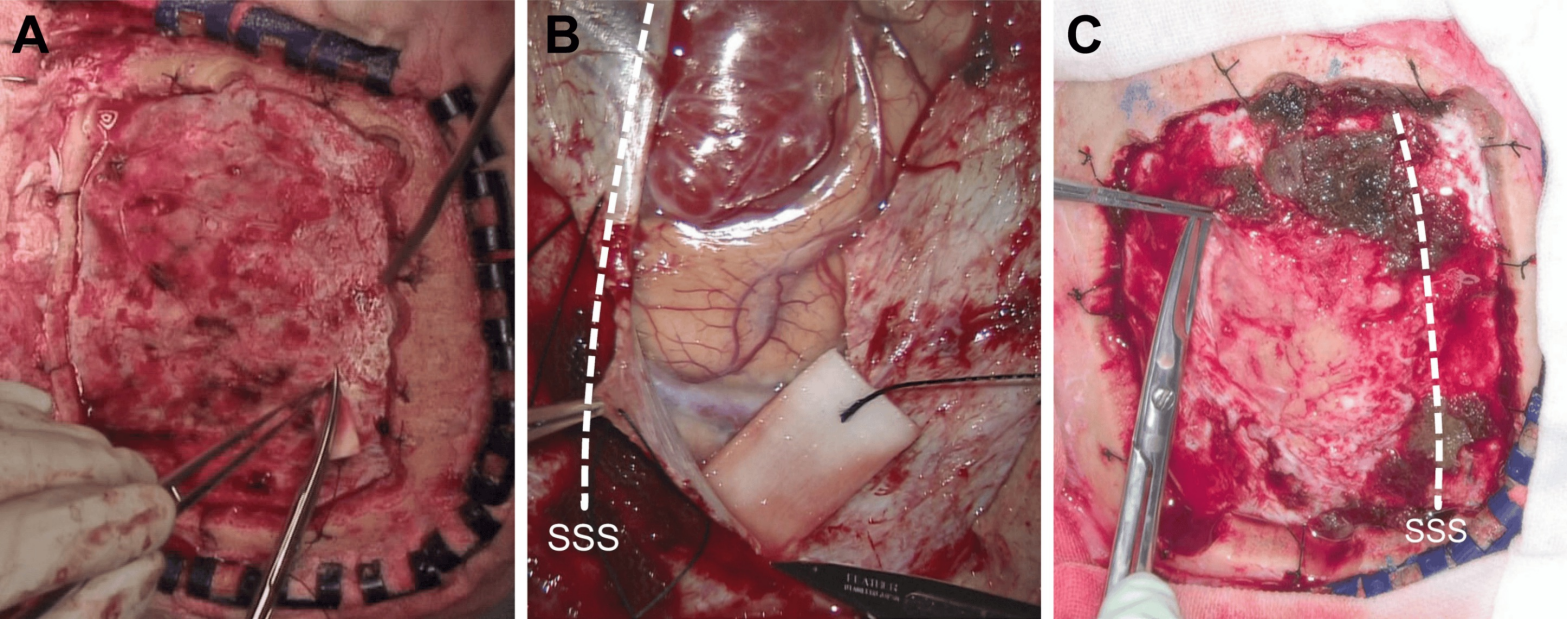
View of the dural incision for meningioma resections. (A) A macroscopic view of the dural incision to resect a right parietal convexity meningioma in a sample case. This photo was taken with a camera attached to a surgical light. (B) A microscopic view of the dural incision to resect a right parasagittal-falx meningioma using a conventional microscope (Leica M525 OH4; Leica Microsystems, Wetzlar, Germany) in another sample case. The surgeon can incise the dura by paying attention to critical structures such as bridging veins, however, the entire area for dural incision cannot be observed. (C) A view of the dural incision to resect a left convexity-parasagittal meningioma using a 4K 3D exoscope in case 2. The entire incised dura can be observed, and critical structures can be magnified when they are located close to the incision. SSS, superior sagittal sinus; 3D, three-dimensional

For superficial meningiomas, the 4K 3D exoscope was considered superior to the conventional microscope (score: +1). Placing its camera away from the surgical field allowed the use of weaker magnification and visualization of a wider surgical field relative to the conventional microscope (Figure 3). When incising the dura close to the critical structure using the exoscope, it provided a magnified view, similar to that provided by the conventional microscope.

**Figure 4:**
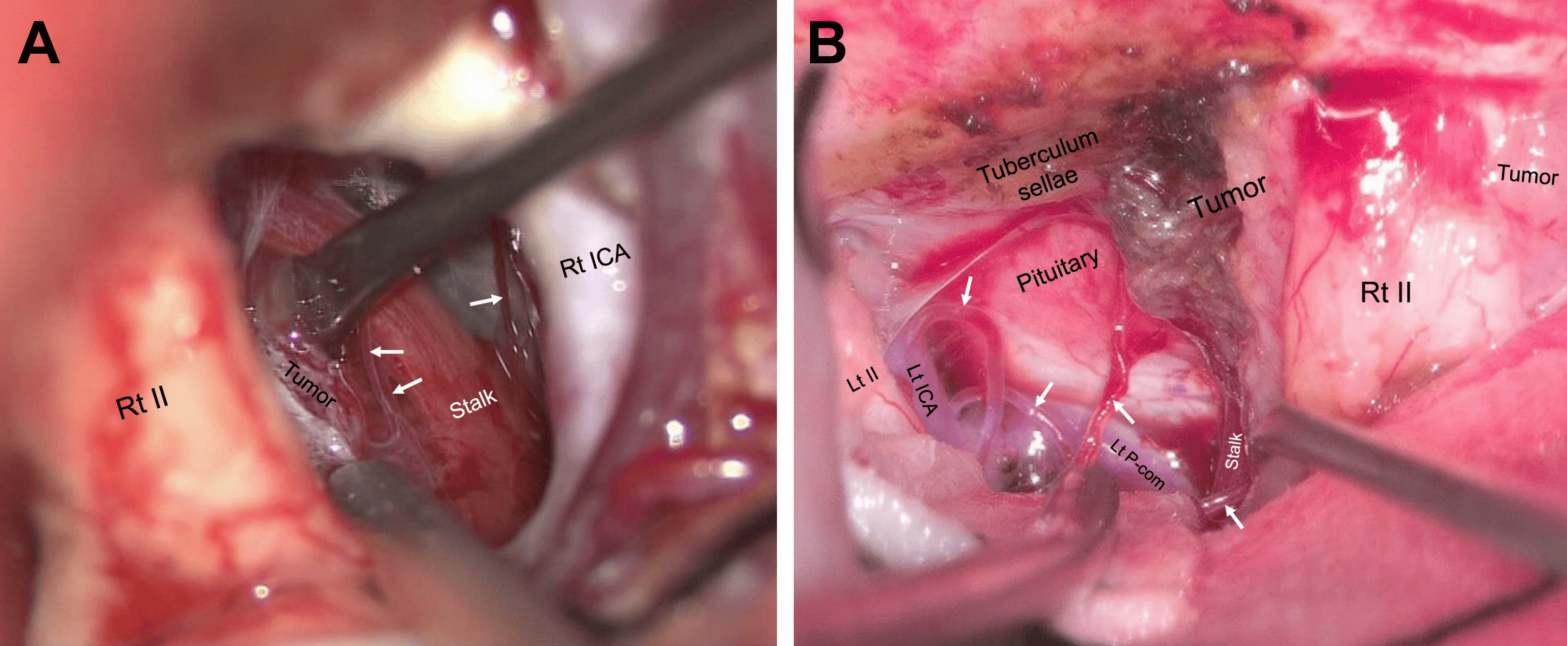
Visibility of deeply located fine structures during tuberculum sellae meningioma resections. (A) A view of the deep structures during surgery in an exemplar case of a tuberculum sellae meningioma resected through the right transsylvian approach using a conventional microscope (Leica M525 OH4). The pituitary stalk and superior hypophyseal arteries (arrows) can be observed between the right optic nerve and internal carotid artery. (B) A view of the deep structures during the surgery for case 7 in the presence of a tuberculum sellae meningioma resected through a right transsylvian approach using a 4K 3D exoscope. The superior hypophyseal arteries (arrows), left posterior communicating artery, and pituitary stalk can be observed between the optic nerves as clearly as with a conventional microscope. II, optic nerve; ICA, internal carotid artery; Lt, left; P-com, posterior communicating artery; Rt, right; Stalk, pituitary stalk; 3D, three-dimensional

For the deeply located tuberculum sellae meningioma, the 4K 3D exoscope was considered equivalent to the conventional microscope (score: 0). Deeply located fine, critical structures (superior hypophyseal arteries, perforators of posterior communicating arteries, and pituitary stalk) could be observed in a bright surgical field using the exoscope as clearly as when using the conventional microscope (Figure 4; Video 1).

**Video 1:** A 51-year-old female patient with a right tuberculum sellae meningioma (case 7). The tumor was resected through the right transsylvian approach using a 4K 3D exoscope. I, olfactory nerve; II, optic nerve; III, oculomotor nerve; Ant, anterior; ICA, internal carotid artery; Lt, left; P-com, posterior communicating artery; Rt, right; SHA, superior hypophyseal artery; Stalk, pituitary stalk; TS, tuberculum sellae; 3D, three-dimensional

Comfort of the surgeon’s arm posture

The 4K 3D exoscope was considered superior to the conventional microscope for all meningiomas included in this study (score: +1), except for a convexity meningioma that could be resected without tilting the microscope (score: 0). When looking up at the target, the conventional microscope must be tilted, which places the surgeon's arms in a fatiguing, extended position and interferes with fine manipulations. The exoscope enabled the surgeon to resect a tumor in a comfortable, flexed arm posture, regardless of the vertical insertion angles of its camera.

Surgeon’s head orientation

**Figure 5:**
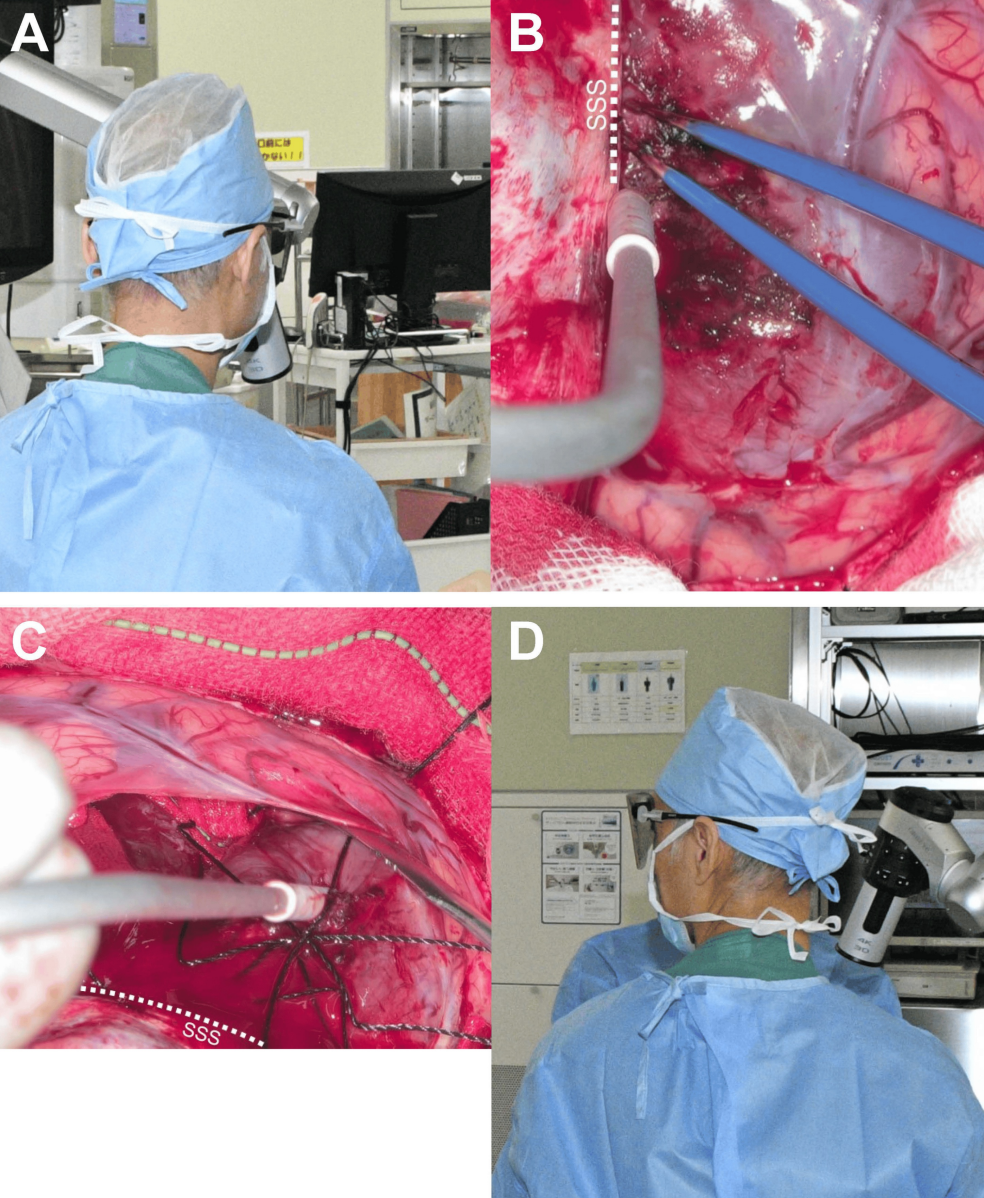
Surgeon’s head orientation during the surgery using a 4K 3D exoscope for case 3 in the presence of a right frontal parasagittal-falx meningioma. (A)-(B) During the tumor detachment from the parasagittal dura and falx, the surgeon can operate while observing the front main monitor in a straight line of sight, with the horizontal direction of the surgeon’s manipulation and gaze coinciding. (C)-(D) During exposure of the postero-lateral tumor surface from the surrounding brain tissue, the surgeon stands on the patient’s left side with an exoscope camera and must proceed with the surgical manipulation with the head turned to the left to watch the main monitor. SSS, superior sagittal sinus; 3D, three-dimensional

Even though the surgeon changed the standing position during surgery, the same exoscope monitor was observed to continue the meningioma resection. Since the large main monitor was difficult to move, the directions in which the surgeon approached the target lesion and observed the main monitor did not necessarily align. A surgeon’s hands and eyes naturally face the same horizontal direction during surgeries when using a conventional microscope; however, this is not necessarily the case when using the exoscope. During the resection of an anterior fossa or a tuberculum sellae meningioma using the exoscope, the direction discrepancy between the surgeon’s manipulation and gaze was not a concern (score: 0), since the directions to insert the camera were limited. However, during the resection of convexity, parasagittal, or middle fossa meningiomas, the exoscope was considered inferior to the conventional microscope (score: -1). Since the exoscope camera could be inserted in various directions to the target lesion, the surgeon could frequently change the camera insertion direction and standing position simultaneously during resection of the tumor. As a result, the surgeon occasionally had to proceed with tumor resection while turning his head toward the side, which was considered a disadvantage of the exoscope (Figure 5; Video 2).

**Video 2:** A 62-year-old male patient with a right frontal parasagittal-falx meningioma (case 3). The tumor was resected using a 4K 3D exoscope with the patient positioned supine on the operating table. Rt, right; 3D, three-dimensional

Perception of the image delay

No time delays between the surgeon’s manipulations and associated 3D images on the monitor were perceived in any of the seven meningioma surgeries using the 4K 3D exoscope (score: 0).

Depths of meningiomas and exoscope suitability

The total scores for each tumor depth, presented in Table 2, depict that the use of the exoscope was superior to that of the conventional microscope for the resection of superficial and deep meningiomas (total score: +0.67, +1) and equivalent for the resection of intermediate meningiomas (total score: +0.33). Overall, the use of the exoscope was suitable for the resection of meningiomas of all depths in this case series (total score: +2).

## Discussion

The major advantage of exoscopic microsurgery over conventional microscopic microsurgery is the surgeon’s ergonomic comfort during the operation [1-4]. Another advantage is the sharing of 3D microsurgical images. Many participants can visualize the same 3D images as the primary surgeon through a monitor with 3D glasses, which contributes to team surgery and the education of surgeons, residents, and medical students [2-4]. In addition to these advantages, a novel 4K 3D exoscope provides 4K-resolution 3D images through which fine structures are clearly visualized [5-10]. Furthermore, its compact design allows easier setup, including transportation, installation in a small space, and draping, and reduces interference with the surgeon's view and manipulation relative to conventional microscopes or other exoscopes [5-10].

To our knowledge, only three case series have mentioned the use of the exoscope during intracranial meningioma surgeries [13-15]. Kijima et al. [13] reported that the exoscope (ORBEYE) was ergonomically more favorable than conventional microscopes in gravity-assisted brain retraction surgery for five supratentorial midline tumors, including four falcine meningeal tumors. Lin et al. [14] reported four cases of successful resection of atrial intraventricular meningiomas using an integrated tubular retraction system with neuro-navigated exoscopes (Karl Storz Endoscopy; Tuttlingen, Germany, or Synaptive Medical, Toronto, Canada). Watanabe et al. [15] reported good outcomes in terms of craniotomy size, blood loss, and surgical time among 34 cases of intracranial meningiomas resected through a combined exoscopic and endoscopic keyhole approach using 2D or 3D exoscopes (Storz VITOM, Tuttlingen, Germany). We evaluated exoscopic meningioma resection from the surgeon’s point of view, especially with regard to the visibility of the surgical field, comfort of the surgeon’s arm posture, surgeon’s head orientation, and perception of the image delay while accounting for the tumor depth.

Visibility of the surgical field

Before using the exoscope, we speculated it to be an intermediate tool between a loupe and a microscope, or to combine features of both. Several neurosurgeons have reported the utility of the 4K 3D exoscope for surgeries of superficial lesions, traditionally performed with a loupe or a microscope, including carotid endarterectomies [16,17] and peripheral nerve surgeries [18,19]. During the resection of a superficial, large meningioma with a conventional microscope, only a small part of the tumor and its surrounding tissues can be visualized because a conventional microscope cannot provide low magnification. However, when using the exoscope, lower magnification can be used to visualize wider surgical fields during the resection of superficial meningiomas by placing the camera farther from the surgical field. The long focal length of the 4K 3D exoscope (220-550 mm) and the flexibility of its camera placement provide a wider field of view (7.5-171 mm) than that provided by a conventional microscope [5,11]. The exoscope could serve as a loupe as well as a microscope and is considered superior to the conventional microscope in terms of the visibility offered during the resection of superficial meningiomas.

We had been skeptical about the exoscope’s visibility for deeply located lesions and their surrounding structures prior to using it. Some neurosurgeons had commented on difficulties during exoscopic surgeries due to differences in depth perception compared with the use of conventional microscopes [11,12] or endoscopes [20], or due to excessively accentuated stereoscopic views in deep surgical fields [21]. When using a 4K 3D exoscope during the tuberculum sellae meningioma resection in the current study, we could observe deeply located fine structures as clearly as with the conventional microscope, without any negative impact on depth perception or stereoscopy. A digital zoom [21], a light-emitting diode source offering a more accurate color contrast than halogen [2], and 4K-resolution 3D imaging quality are thought to enable the depiction of anatomical details even in deep surgical fields. Some neurosurgeons have reported the utility of the 4K 3D exoscope during microsurgery for deep lesions, such as the retrosigmoid approach for cerebellopontine angle lesions [22-24] and transsphenoidal pituitary surgery [25]. Moreover, the easy and safe concomitant use of the endoscope is an advantage of the exoscope; the endoscope can be inserted easily and moved safely while looking at the exoscopic and endoscopic screens simultaneously [23].

Comfort of the surgeon’s arm posture

Almost all literature relating to exoscopic surgery describes the surgeon’s ergonomics as superior to those in conventional microscopic surgery. From our perspective, the surgeon’s arm posture is more important than the surgeon’s head-up posture. During manipulation in the 12 o’clock direction with a conventional microscope, surgeons must extend their arms excessively [26]. Operating with extended arms can easily cause fatigue and make it difficult to perform fine manipulations. During meningioma surgeries, surgeons occasionally tilt the microscope and extend their arms. However, the exoscope allows the surgeon to adopt a comfortable, flexed arm posture regardless of the vertical insertion angles of its camera, resulting from the surgeon's freedom from microscopic ocular lenses. Some neurosurgeons have reported the usefulness of the exoscope in various neurosurgical procedures that required arm extension or an unnatural posture associated with the use of the conventional microscope [13,22,27,28].

Surgeon’s head orientation

While using the conventional microscope, surgeons stand facing the target lesion and direct the microscope toward the lesion. The directions of the surgeon’s manipulation and gaze are identical. While using the exoscope, its camera, corresponding to a microscope’s objective lens, could be inserted from various directions; however, its large 3D monitor, corresponding to a microscope’s ocular lens, was difficult to move. The camera always faced a similar direction relative to the surgeon, although the associated monitor was not necessarily in the direction the surgeon was facing. Therefore, the surgeon occasionally had to operate with his head turned to the side to watch the main monitor, especially for lesions that could be approached from various angles, such as meningiomas located superficially or within the middle cranial fossa. Assistant surgeons found it more difficult to contribute to the surgery. Several neurosurgeons have reported impaired hand-eye coordination as a disadvantage of the exoscope [8,11,20]. We consider the unnatural head orientation during operations and the difficulty for assistant surgeons to contribute to the procedure to be disadvantages of the exoscope. In the future, developments in 3D monitor technology are expected, such as using multiple monitors or small 3D goggles.

Perception of the image delay

Some neurovascular surgeons within our department have experienced manipulation difficulties during bypass surgery using the 4K 3D exoscope due to their perception of a subtle delay between their manipulations and the corresponding 3D images on the monitor and had to revert to the use of a conventional microscope to continue the surgery. However, such image latency was not perceived during any of the present meningioma surgeries. In two previous studies on bypass surgery using the exoscope, one reported excellent visualization of the delicate vessel anatomy and ease of microanastomosis without any mention of image delays [29], and the other reported a higher number of unnecessary movements and longer bypass duration during experimental microanastomoses using the 4K 3D exoscope compared to those using other exoscopes, although the specific underlying reason was not identified [30]. We believe that neurosurgeons do not perceive a subtle image delay in most microsurgical procedures other than bypass surgeries and that such perceptual issues may be resolved with a surgeon’s increasing experience in using the exoscope.

Limitations

The main limitation of this study is the small number of patients, especially those with deep meningiomas. This could be addressed in future studies performed in a larger study population across multiple surgeons and centers, with the collection of structured feedback data.

## Conclusions

The 4K 3D exoscope was suitable for operations on meningiomas of all depths. It allowed the surgeon to observe both the wide, superficial surgical field and the clear, deep structures, and to operate comfortably on meningiomas of all depths with arms flexed. However, the surgeon occasionally had to operate with his head turned to the side when using the exoscope because of the immobility of its monitor, despite the wide availability of its camera insertion from various directions to access meningiomas located superficially or within the middle cranial fossa. The discrepancy between the surgeon’s manipulation and gaze directions was a disadvantage of the exoscope. Further technological developments in the 3D monitor are expected to resolve this issue in the future.
